# 

**Published:** 1909-06

**Authors:** 


					CONTENTS. Page
Small-pox in Bristol and Neighbourhood (1908-9).
By D. S.
Davies, M.D. Lond.
The Indications for Gastro - Enterostomy.
By T. Carwardine,
M.S. Lond., F.R.C.S.
107
A Family with a Congenital Deformity of the Nose, and the
Results of Subcutaneous Injection ot Wax.
i3y h.. Jrl. E. Stack,
M.B., B.C., F.R.C.S. ...
Ir9
A Case of Lead Encephalopathy.
By J. Angell James, M.R.C.S.,
L.R.C.P.
124
Cretinism and the Puerperium: Sequel to a Recorded History.
By David A. Alexander, M B.
128
A Discussion on "Treatment by lonisation or Cataphoresis."
Opened by H. P. Tayler, M.A., M.B. Cantab., and H. Lewis Jones,
M.A., M.D.Cantab., F.R.C.P
132
Electricity in General Practice.
By Herbert P. Tayler, M.A.,
M B. Cantab.
145
Progress of the Medical Sciences :
Medicine.
J. Michell Clarke, M.A., M.D., F.R.C.P.
152
Surgery.
C. A. Morton, F.R.C.S.
157
Dermatology.
J. A. Nixon, B.A., M.B., M.R.C.P.
i6i
Reviews of Books
i&5
Editorial Notes
178
Notes on Preparations for the Sick
183
The Library of the Bristol Medico-Chirurgical Society   184
Meetings of Societies
186
Local Medical Notes
i?9

				

